# Neuropathological correlates of parkinsonian disorders in a large Dutch autopsy series

**DOI:** 10.1186/s40478-020-00914-9

**Published:** 2020-03-26

**Authors:** H. Geut, D. H. Hepp, E. Foncke, H. W. Berendse, J. M. Rozemuller, I. Huitinga, W. D. J. van de Berg

**Affiliations:** 1 Department of Anatomy and Neurosciences, Amsterdam Neuroscience, Amsterdam UMC, Vrije Universiteit Amsterdam, P.O. Box 7057, 1007 MB Amsterdam, the Netherlands; 2 Netherlands Brain Bank, Netherlands Institute for Neuroscience, Amsterdam, the Netherlands; 3 Department of Neurology, Amsterdam Neuroscience, Amsterdam UMC, Vrije Universiteit Amsterdam, Amsterdam, the Netherlands; 4 Department of Pathology, Amsterdam Neuroscience, Amsterdam UMC, Vrije Universiteit Amsterdam, Amsterdam, the Netherlands

**Keywords:** Parkinson’s disease, Lewy pathology, Amyloid-β

## Abstract

The clinical diagnosis in patients with parkinsonian disorders can be challenging, and a definite diagnosis requires neuropathological confirmation. The aim of this study was to examine whether a clinical diagnosis of Parkinson’s disease (PD) and atypical parkinsonian disorders predict the presence of Lewy pathology (LP) and concomitant neuropathological lesions.

We included 293 donors with a history of parkinsonism without dementia at disease onset, collected by the Netherlands Brain Bank (NBB) from 1989 to 2015. We retrospectively categorized donors according the International Parkinson and Movement Disorder Society clinical diagnostic criteria for PD (MDS-PD criteria) as ‘not PD’, ‘probable PD’ or ‘established PD’. We compared the final clinical diagnosis to presence of neuropathological lesions as defined by BrainNet Europe and National Institute on Aging – Alzheimer's Association guidelines.

LP was present in 150 out of 176 donors (85%) with a clinical diagnosis of PD, in 8 out of 101 donors (8%) with atypical parkinsonian disorders and in 4 out of 16 donors (25%) without a definite clinical diagnosis. Independent from age at death, stages of amyloid-β, but not neurofibrillary tau or neuritic plaques, were higher in donors with LP compared to other types of pathology (*p* = 0.009). The MDS-PD criteria at a certainty level of ‘probable PD’ predicted presence of LP with a diagnostic accuracy of 89.3%. Among donors with LP, ‘established PD’ donors showed similar Braak α-synuclein stages and stages of amyloid-β, neurofibrillary tau and neuritic plaques compared to ‘not PD’ or ‘probable PD’ donors.

In conclusion, both a clinical diagnosis of PD as well as MDS-PD criteria accurately predicted presence of LP in NBB donors. LP was associated with more widespread amyloid-β pathology, suggesting a link between amyloid-β accumulation and LP formation.

## Introduction

The core motor symptoms of Parkinson’s disease (PD) include bradykinesia, rigidity and tremor. Together, these symptoms form a clinical syndrome known as parkinsonism. Parkinsonism can be present in other clinical conditions, among others, multiple system atrophy (MSA), progressive supranuclear palsy (PSP), corticobasal syndrome (CBS) and vascular parkinsonism [1].

The main neuropathological hallmarks of PD include nigral degeneration, combined with Lewy bodies (LBs) and Lewy neurites, together termed Lewy pathology (LP). However, LP has been found in only 71–90% of clinically defined PD patients [2–7]. The accuracy of a clinical diagnosis of PD to predict the presence of LP is dependent on the time of diagnosis after disease onset [2], whether or not the diagnosis was made by a movement disorders expert [8], and the diagnostic criteria used [8]. Moreover, LP has been observed in patients with a wide variety of other clinical diagnoses [2, 3, 6, 7, 9, 10].

The difficulty in discriminating between the different parkinsonian disorders based on the clinical phenotype may on the one hand be attributed to overlap of symptoms and signs between the different disorders, and on the other hand to clinical heterogeneity within these disorders. The clinical heterogeneity of PD is well-known, and different clinical subtypes of PD have been proposed [11, 12]. Similarly, atypical parkinsonian disorders are also clinically heterogeneous entities [13].

Another factor that complicates the discrimination between different parkinsonian disorders is the presence of concomitant pathology. Mixed pathology is common in neurodegenerative diseases, especially in aged individuals [14, 15]. In particular, LP in brains of PD donors is often accompanied by Alzheimer’s disease (AD) type pathology including amyloid-β positive plaques, tau neurofibrillary pathology and neuritic plaques. Several autopsy studies in PD donors have found a relationship between dementia and the load of cortical LP [16, 17] as well as the load and distribution of AD-type pathology [16–18]. Therefore, the presence and load of concomitant pathology is considered as a factor underlying clinical heterogeneity in parkinsonian disorders [8, 14].

In the current study among a large postmortem cohort of brain donors with parkinsonian disorders collected by the Netherlands Brain Bank (NBB), we aimed to examine whether a clinical diagnosis of PD or atypical parkinsonian disorders predict the presence of LP and concomitant neuropathological lesions. Specifically, we studied the relation between the clinical diagnosis and the presence of neuropathological hallmarks as well as the level of concomitant AD-type pathology. Second, we determined the accuracy of the clinical diagnosis of PD according to standardized clinical criteria to predict the presence of LP. Third, among donors with LP, we studied the association between standardized clinical criteria for PD and Braak α-synuclein stages as well as stages of AD-type pathology.

## Methods

### Donor selection

Clinical records of all donors (*n* = 4003) collected by the NBB in Amsterdam, the Netherlands, from 1989 to 2015 were screened for inclusion in this study. Residents from the Netherlands can register themselves as a brain donor at the NBB since 1985. For all donors, a written informed consent for brain autopsy and the use of the material and clinical information for research purposes had been obtained from the donor or the next of kin.

Inclusion criteria for the current study were: 1) the presence of parkinsonism during the course of disease, defined as bradykinesia in combination with rigidity or tremor [19], 2) availability of clinical information on clinical diagnosis, disease duration and presence and timing of parkinsonism and dementia, 3) autopsy and neuropathological examination according to standardized protocols by the NBB (open access: www.brainbank.nl), and 4) no dementia before or within 1 year after the onset of parkinsonism. The presence of LP in donors with dementia within the first year of disease onset by definition leads to the diagnosis of dementia with Lewy bodies (DLB) based on clinical criteria [20]. As neuropathological examination was used as gold standard in this study to determine diagnostic accuracy, and DLB cannot be distinguished from PD with dementia based on neuropathological findings, DLB donors were excluded from this study based on the presence of dementia within the first year of disease onset. As a consequence, all donors with parkinsonism in the context of an existing dementia were excluded, so this study comprises donors with primarily a movement disorder.

Clinical summaries were searched using the key terms “parkinson”, “extra-pyramidal”, “tremor”, “bradykinesia”, “hypokinesia”, “akinesia”, “rigidity”, “cogwheel”, including synonyms and spelling variants. The 1022 clinical summaries with at least one of these key words were manually searched for the presence of parkinsonism. We identified 591 donors with parkinsonism during the course of disease. Clinical information was insufficient in 42 donors, including 28 donors with LP and nigral degeneration. In 6 donors, no standardized neuropathological assessment had been performed. Of the other 543 donors, 250 donors presented with dementia before or within 1 year after the onset of parkinsonism, including 85 donors with LP and nigral degeneration. The remaining 293 donors were included in this study.

### Clinical information

Medical files were requested from the treating physicians at the time of autopsy. Information was abstracted from the clinical files by an assessor blinded to the neuropathological findings (HG). The recorded clinical information included: sex, age at onset, age at death, disease duration from time at onset of first symptoms, presence and timing of parkinsonism and dementia. When available, information was recorded on presence of visual hallucinations, a history of parkinsonism in first-degree or second-degree relatives, presence of bradykinesia, rest tremor, rigidity, clear response to dopaminergic treatment, presence of olfactory loss, and presence of atypical symptoms (exclusion criteria or so-called ‘red flags’) during the course of disease as defined by the International Parkinson and Movement Disorder Society (MDS) clinical diagnostic criteria for PD (MDS-PD criteria) [19]. Furthermore, initial clinical diagnosis within 1 year after disease onset was recorded. Lastly, the final clinical diagnosis was reported by the treating physician upon admission to the NBB after death of the donor, but before the brain autopsy findings were known. The final clinical diagnosis was determined by a movement disorders specialist in 40% (*n* = 117), by a general neurologist in 40% (*n* = 117), or by another type of physician, i.e. general practitioner, psychiatrist or geriatrist in 6% (*n* = 19). In 14% (*n* = 40) the specialization level of the physician who determined the diagnosis was unknown.

Donors were classified based on their final clinical diagnosis as PD, atypical parkinsonian disorders or no definite clinical diagnosis. The atypical parkinsonian disorders group comprised four diagnostic categories: 1) MSA, 2) PSP, 3) vascular parkinsonism, and 4) other clinical diagnoses.

To study the association between the clinical classification according to standardized diagnostic criteria for PD and pathological stages, MDS-PD criteria were retrospectively applied. Clinical information was considered sufficient when 1) the donor payed regular visits to a neurologist during at least the first 5 years after first presentation, or until death when disease duration was less than 5 years, and 2) a detailed history and neurological examination was documented on at least one occasion when an absolute exclusion criterion was present, or on at least three occasions during the disease course when absolute exclusion criteria were not mentioned. This information was available for 234 donors, resulting in a classification of each of these donors as ‘not PD’, ‘probable PD’ or ‘established PD’ [19].

### Neuropathological assessments

Autopsy was performed using a standardized protocol by the NBB (open access: www.brainbank.nl). Post-mortem examination was performed by two experienced neuropathologists (JR and WK). The presence of neuropathological hallmarks was assessed following consensus criteria for diagnosis of PD [21], MSA [22], PSP [23], AD [24], frontotemporal dementia (FTD) and corticobasal degeneration (CBD) [25]. LP was defined as the presence of Lewy bodies and Lewy neurites. Donors were diagnosed with vascular parkinsonism when either vascular lesions were present in nigrostriatal regions, or features of small vessel disease, such as enlargement of perivascular spaces, perivascular pallor, gliosis or hyaline thickening of the vascular walls, were present in periventricular regions, in the absence of other neuropathological lesions apart from age-related changes. Thal amyloid-β phase, Braak stage for neurofibrillary (NFT) pathology and CERAD neuritic plaque scores were determined according to the most recent National Institute on Aging – Alzheimer’s Association guidelines [24], and Braak and McKeith stages for LP were determined according to the BrainNet Europe guidelines [26].

### Statistical analysis

Differences in demographics and clinical features between PD and atypical parkinsonian disorders, between LP and other types of pathology and between donors with LP fulfilling MDS-PD criteria for ‘established PD’ and ‘not PD’ combined with ‘probable PD’, were compared using t-tests for continuous variables and chi-square tests for categorical variables.

Braak α-synuclein stage, Thal amyloid-β phase, Braak NFT stage and CERAD neuritic plaque scores were compared between donors with different clinical diagnoses, i.e. PD, PSP, MSA and other clinical diagnoses, and between donors with different types of pathology, i.e. LP, tau pathology typical for PSP (PSP tau), glial cytoplasmic inclusions (GCIs) and other types of pathology. For these comparisons, we used analysis of covariance including age at death as covariate. When the omnibus test for type of pathology was significant, planned contrasts were used to compare the PD or LP group to the other groups. To further analyze the relation between pathological diagnosis and the level of AD-type pathology, a second analysis was done including both age at death and presence of dementia as covariates in the model. In donors with LP, neuropathological stages were compared between donors fulfilling MDS-PD criteria for ‘established PD’ and ‘not PD’ combined with ‘probable PD’, using analysis of covariance including age at death as covariate in the model.

Among the 234 donors with sufficient clinical information for applying the MDS-PD criteria, sensitivity was calculated as the percentage of donors with a clinical diagnosis of PD within the group of donors with LP. Specificity was calculated as the percentage of donors without a clinical diagnosis of PD within the group of donors without LP. Overall diagnostic accuracy was defined as the percentage of donors with a neuropathologically confirmed clinical diagnosis, i.e. clinical PD donors with LP plus donors without LP and without a clinical PD diagnosis, within the total of 234 donors. All analyses were run on SPSS version 26.

## Results

### Demographics and clinical characteristics

A total of 293 NBB donors with clinically primarily a parkinsonian movement disorder were included in the study. Of all donors, 60% were male. Mean age at onset was 63 ± 11 years, mean disease duration was 12 ± 8 years and mean age at death was 75 ± 9 years.

A final clinical diagnosis of PD was made in 176 donors (60%). Other clinical diagnoses included PSP (*n* = 40), MSA (*n* = 29), vascular parkinsonism (*n* = 14), drug-induced parkinsonism (*n* = 6), CBS (*n* = 5), AD (*n* = 2), FTD, multiple sclerosis, Fahr’s syndrome, amyotrophic lateral sclerosis and polymyalgia rheumatica (all *n* = 1). These donors were categorized as the ‘atypical parkinsonian disorders’ group. Sixteen donors did not receive a definite clinical diagnosis and were analyzed as a separate clinical group. The specialization level of the diagnosing physicians was similar between different clinical groups, but the atypical parkinsonian disorders group was assessed on average in a more recent year than the PD group (2002 vs. 1995, *p* < 0.001; Table 1).

**Table 1:** Demographics and clinical characteristics of donors with clinically-defined PD, atypical parkinsonian disorders and donors without a definite clinical diagnosis.

	PD	Atypical parkinsonian disorders	No definite clinical diagnosis
N	176	101	16
Sex *M*, n (%)	104 (59%)	61 (60%)	11 (69%)
Age at onset *y,* mean ± SD (range), <50 / 50-75 / >75 y, n (%)	62 ± 11 (32–88), 26/129/21 (15/73/12%)	63 ± 9 (36–81), 8/84/9 (8/83/9%)	66 ± 14 (34–84), 2/10/4 (13/63/25%)
Age at death *y*, mean ± SD (range)	77 ± 8 (56–98)	72 ± 9 (42–92)***	77 ± 11 (54–88)
Disease duration *y*, mean ± SD (range)	15 ± 8 (2–42)	8 ± 6 (0.5–38)***	11 ± 9 (3–41)*
Dementia, n (%)	90 (51%)	39 (39%)*	4 (25%)*
Duration of dementia *y*, mean ± SD (range)	4 ± 3 (0–12)	2 ± 2 (0–10)***	4 ± 2 (2–5)
Visual hallucinations, n/N (%)	95/121 (79%)	22/28 (79%)	0/6 (0%)***
Rest tremor, n/N (%)	120/143 (84%)	38/68 (56%)***	10/14 (71%)
Clear response to dopaminergic therapy, n/N (%)	118/135 (87%)	12/55 (22%)***	3/12 (25%)***
Positive family history of parkinsonism, n/N (%)	33/86 (38%)	14/60 (23%)	2/8 (25%)
APOE ε4 alleles, n/N (%)			
0	78/115 (68%)	48/80 (60%)	9/11 (82%)
1	33/115 (29%)	29/80 (36%)	2/11 (18%)
2	4/115 (3%)	3/80 (4%)	0/11 (0%)
Specialization level of the diagnosing physician, n/N (%)			
Movement disorders specialist	59/144 (41%)	52/95 (55%)	6/14 (43%)
General neurologist	76/144 (53%)	37/95 (39%)	4/14 (29%)
Non-neurologist	9/144 (6%)	6/95 (6%)	4/14 (29%)**
Year of diagnosis, mean ± SD (range)	1995 ± 10 (1970 – 2012)	2002 ± 6 (1988 – 2014)***	1998 ± 10 (1979 – 2013)

*P*-values are shown for comparisons between the PD group and the atypical parkinsonian disorders group or the group without a definite clinical diagnosis using t-tests for continuous variables and chi-square tests for categorical variables. * *p* < 0.05; ** *p* < 0.01; *** *p* < 0.001

Of the 176 clinically-defined PD donors, 59 donors (34%) presented with a tremor-dominant clinical phenotype. Mean age at onset was equal between the PD and atypical parkinsonian disorders group (62 ± 11 vs. 63 ± 9 years), but mean age at death was higher in the PD group (77 ± 8 vs. 72 ± 9 years, *p* < 0.001) due to a longer mean disease duration in these donors (15 ± 8 vs. 8 ± 6 years, *p* < 0.001). Presence of dementia at time of death was more frequent in the PD group compared to the atypical parkinsonian disorders group (51% vs. 39%, *p* = 0.044), and duration of dementia was longer (4 ± 3 vs. 2 ± 2 years, *p* < 0.001). Visual hallucinations were equally frequent in both groups (79% in both), as was the case for a positive family history of parkinsonism (38% vs. 23%, *p* = 0.056; Table 1).

### A wide range of neuropathological hallmarks was found in clinically-defined PD

**Fig. 1:**
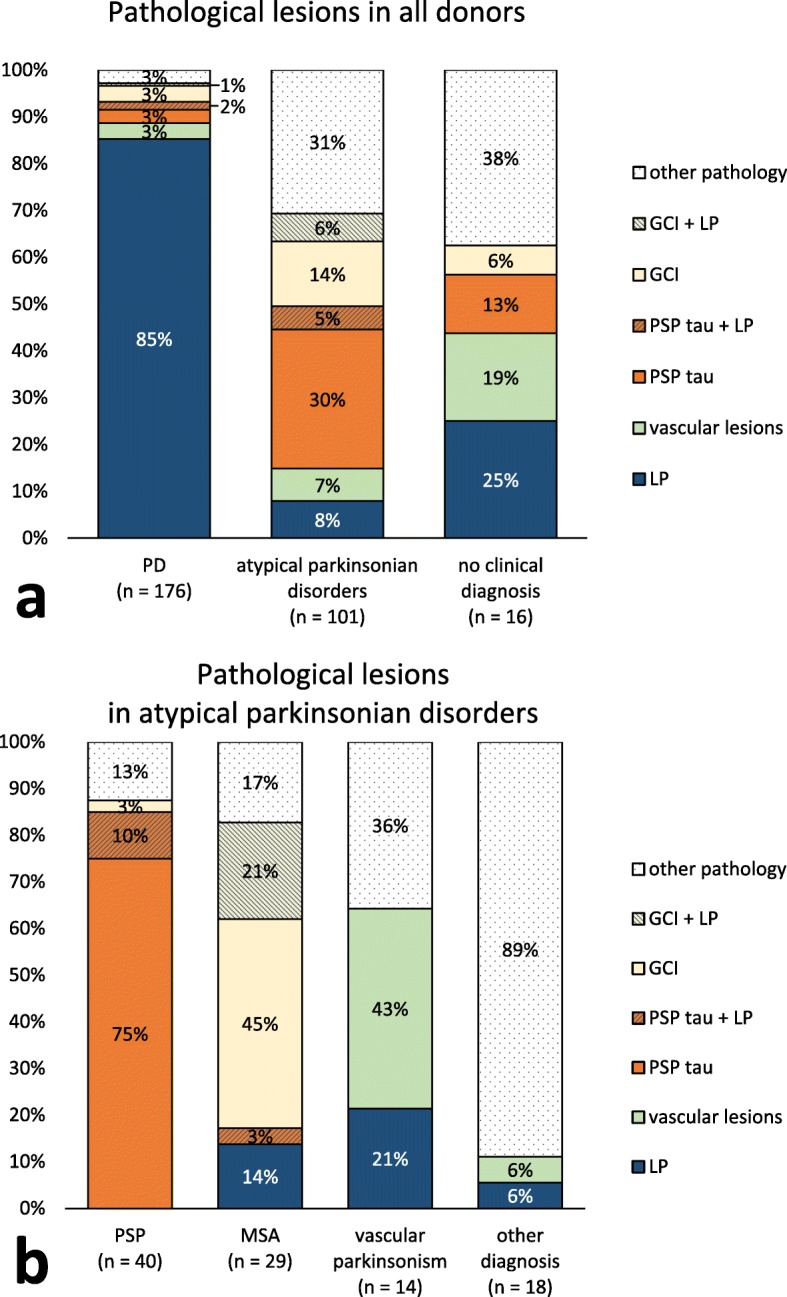
**a.** LP was present in 85% of PD donors, but also in 8% of donors with atypical parkinsonian disorders. Other pathological lesions in PD donors included vascular lesions, PSP tau pathology and GCIs. **b.** Among donors with atypical parkinsonian disorders, LP was present in donors with a clinical diagnosis of MSA, vascular parkinsonism and other diagnoses. Concomitant LP was frequently found in donors with GCI (yellow-blue stripes) or PSP tau lesions (orange-blue stripes). GCI = glial cytoplasmic inclusions; PSP tau = tau pathology typical for progressive supranuclear palsy; LP = Lewy pathology

The pathological correlates of the clinical diagnostic groups were studied by assessing the main pathological hallmarks in the brain. LP as the main neuropathological hallmark was present in 150 out of 176 clinically-defined PD donors (85%), compared to 8 out of 101 donors with atypical parkinsonian disorders (8%) and 4 out of 16 donors with no definite clinical diagnosis (25%; Fig. 1). The clinical diagnoses in the eight donors with LP from the atypical parkinsonian disorders group included MSA (*n* = 4), vascular parkinsonism (*n* = 3) and CBS (*n* = 1).

Among the donors with LP in the PD group, brainstem-predominant LP was present in 7 donors (5%), limbic-transitional LP in 71 donors (47%), diffuse neocortical LP in 43 donors (29%), amygdala-predominant LP in 9 donors (6%) and in 20 donors (13%) insufficient brain regions were available for assessment of LP distribution. Among donors with LP in the atypical parkinsonian disorders group, limbic-transitional LP was present in 4 donors (50%), diffuse neocortical LP in 3 donors (37.5%), and insufficient brain regions were available to assess LP distribution in 1 donor (12.5%).

**Fig. 2:**
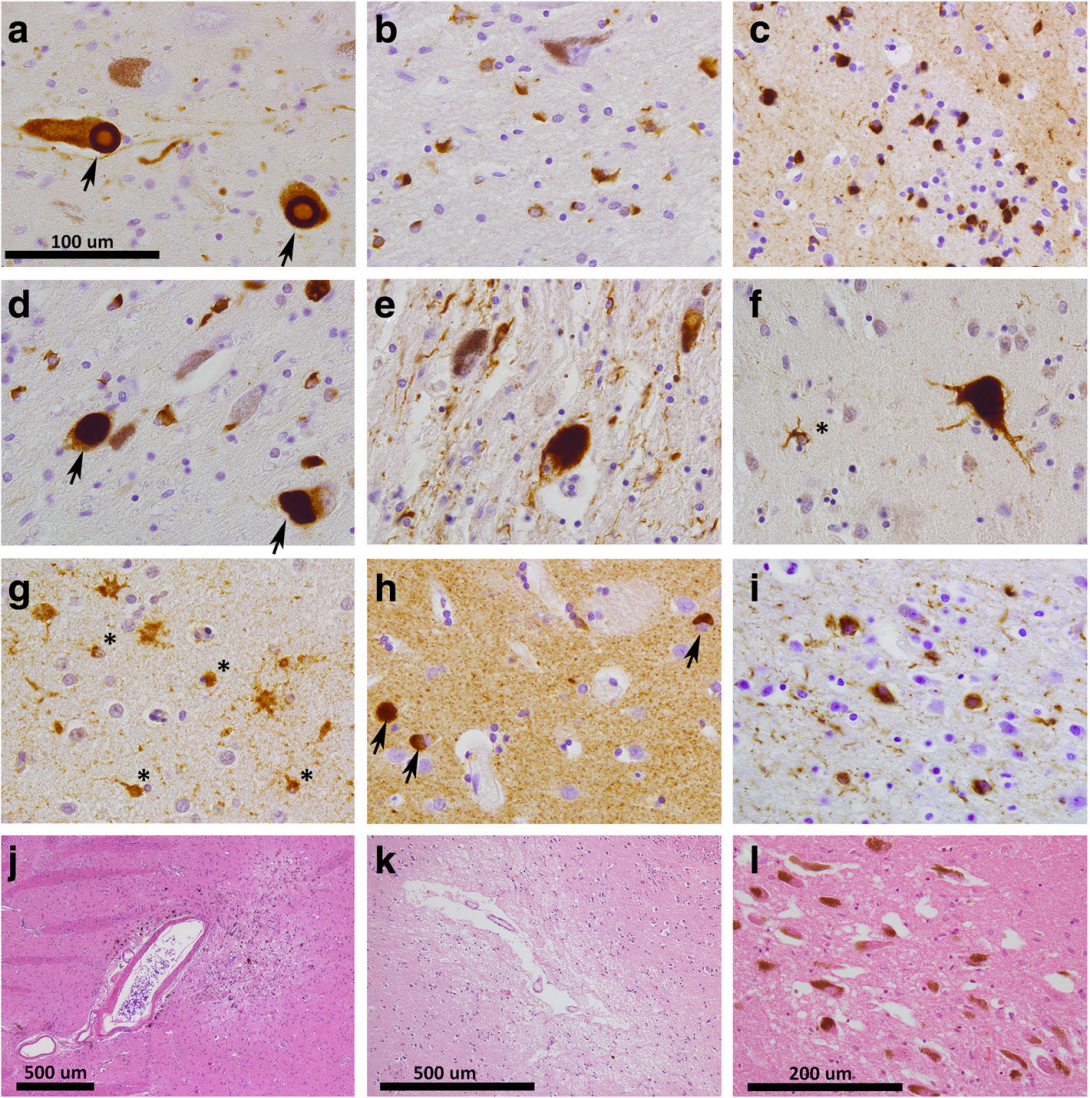
Donors with a clinical diagnosis of PD showed a wide variety of neuropathological hallmarks in nigrostriatal regions. **a**. 85% of clinical PD donors showed nigral degeneration combined with α-synuclein positive Lewy bodies (arrows) and Lewy neurites in the substantia nigra. **b-c**. Α-synuclein positive glial cytoplasmic inclusions (GCIs) in the substantia nigra (**b**) and striatum (**c**), fitting a neuropathological diagnosis of MSA. **d**. Α-synuclein positive GCIs combined with Lewy bodies (arrows) in the substantia nigra. **e-f**. HPF-tau positive globose tangles and neurites in the substantia nigra (**e**) and a globose tangle and coiled body (asterisk) in the striatum (**f**) of a donor with a neuropathological diagnosis of PSP. **g-h**. In the striatum of a donor with combined PSP tau pathology and Lewy pathology, HPF-tau positive coiled bodies (asterisks) and thorny astrocytes (**g**) were present as well as α-synuclein positive Lewy bodies (arrows in **h**). **i**. One donor with early onset PD showed nigral degeneration and gliosis combined with HPF-tau positive pathology in the basal ganglia, thalamus and subthalamic nucleus. Image I shows neuritic threads and neuronal cytoplasmic inclusions in the nucleus basalis of Meynert. **j-k**. Vascular lesions were found in the nigrostriatal regions of several donors, including a donor with a microvascular lesion in the putamen (**j**) and a donor with wide perivascular spaces and a lacunar state of the striatum (**k**). **l**. In one donor with familial tremor-predominant parkinsonism, no protein aggregates or vascular lesions were present. The substantia nigra did not show neuronal loss, but mild spongiform changes were present. The scale bar in **a** applies to images **a-i**

Besides LP, other neuropathological hallmarks in clinically-defined PD donors included PSP tau pathology (*n* = 8, 5%), GCIs indicating a neuropathological diagnosis of MSA (*n* = 7, 4%), vascular lesions in nigrostriatal regions or features of small vessel disease in periventricular areas (*n* = 6, 3%), no neuropathological lesions (*n* = 2, 1%), AD-type pathology (*n* = 2, 1%) and a rare tauopathy (*n* = 1, 1%) (Figs. 1 and 2).

The two donors without neuropathological lesions did not show any significant pathological hallmarks besides mild age-related pathology, and no nigrostriatal neuronal loss. The clinical history of one donor without neuropathological lesions mentioned chronic use of alimemazine and flunarizine as treatment for vestibular vertigo, both of which could have caused the parkinsonism. The other donor without neuropathological lesions had a late-onset, tremor-predominant parkinsonism. The mother and sister of the donor suffered from similar symptoms. The substantia nigra of this donor showed mild spongiform changes, but no protein aggregates or vascular lesions (Fig. 2l).

The parkinsonian symptoms in the donor with a rare tauopathy started at age 44 after a pertussis infection. The family history in this donor is unknown. The motor symptoms were slowly progressive and responded well to levodopa. Over time, stuttering, dystonia, spasticity and an abducens paresis developed, but the donor remained cognitively normal until his death at age 81. Brain autopsy showed neuronal loss and gliosis in the substantia nigra, HPF-tau positive neuronal cytoplasmic inclusions, many neurofibrillary threads and thorny astrocytes in the basal ganglia, thalamus and subthalamic nucleus, but no tufted astrocytes as seen in PSP (Fig. 2i).

### Combinations of LP with PSP tau pathology or GCIs are frequent

**Fig. 3:**
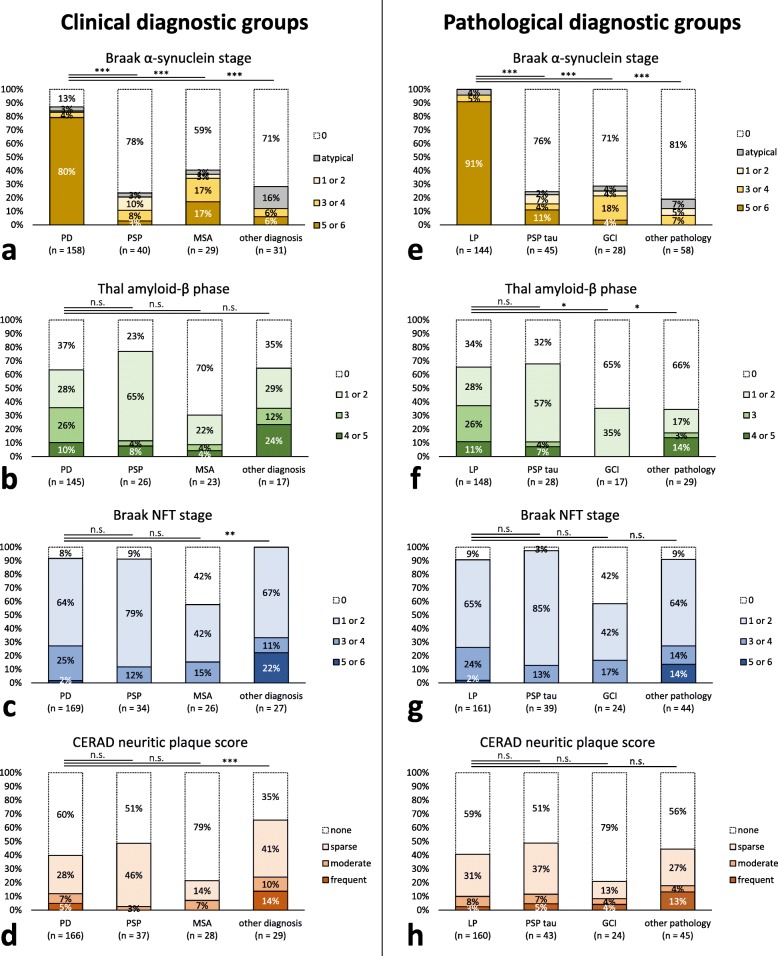
Stages of LP and AD-type pathology in donors grouped according to clinical diagnoses (**a-d**) and pathological diagnoses (**e-h**). **a.** Clinically-defined PD donors showed higher Braak α-synuclein stages than donors with atypical parkinsonian disorders. **b. **Thal amyloid-β phases were not different between clinical diagnostic groups. **c. **Braak NFT stage and **d.** CERAD neuritic plaque score were similar between PD, PSP and MSA donors, but higher in donors with other diagnoses. **e-h.** LP with an amygdala-predominant distribution or at least Braak stage 3 was present in 18% of donors with PSP tau pathology, in 25% of donors with GCI pathology and in 17% of donors with other types of neuropathology. **f.** In donors with LP, Thal amyloid-β phases were higher than in donors with GCI pathology or other pathological lesions, when controlling for age at death. **g,h.** Braak NFT stages (**g**) and CERAD neuritic plaques scores (**h**) were not different between donors with different pathological diagnoses. LP = Lewy pathology; PSP tau = tau pathology typical for progressive supranuclear palsy; GCI = glial cytoplasmic inclusion

We assessed the presence of mixed pathology, as this may complicate the discrimination between different parkinsonian disorders. Combinations of LP (defined as Braak α-synuclein stage ≥3 or Braak atypical stage with amygdala-predominant distribution) with other neuropathological hallmarks were frequently present in both the PD group and the atypical parkinsonian disorders group. A combination of PSP tau pathology and LP was found in 8 out of 45 donors with PSP tau pathology (18%; Figs. 2g-h and 3e). LP distribution was brainstem-predominant in one donor, limbic-transitional in five donors and amygdala-predominant in two donors. Three of these mixed PSP + LP donors were clinically diagnosed as PD, four as PSP and one as MSA. A combination of GCI pathology and LP was present in 7 out of 28 donors with GCIs (25%; Figs. 2d and 3e). The distribution of LP was brainstem-predominant in five donors, limbic-transitional in one donor and diffuse-neocortical in one donor. Six out of seven of these mixed GCI + LP donors were clinically diagnosed as MSA, the other donor was diagnosed as PD.

In donors with other types of pathology, LP was present in 8 out of 58 donors (14%; Fig. 3e). Combined clinicopathological diagnoses in these donors included AD (*n* = 2), FTD (*n* = 1), CBD (*n* = 1), drug-induced parkinsonism (*n* = 1), capillary hemangioblastoma of the medulla oblongata (*n* = 1) and vascular parkinsonism (*n* = 2).

### AD-type pathology is similar in clinically-defined PD, PSP and MSA

To study whether the different clinical phenotypes predicted a different level of concomitant AD-type pathology, we compared the neuropathological stages of AD-type pathology between different clinical diagnostic groups using analysis of covariance including age at death as covariate (Fig. 3a-d). Thal amyloid-β phases were not different between groups (F = 2.2, *p* = 0.09; Fig. 3b). Braak NFT stage and CERAD neuritic plaque score were similar between PD compared to PSP and MSA donors, but higher in donors with other diagnoses (F = 4.5; planned contrasts *p* = 0.004 and F = 3.8; planned contrasts *p* = 0.001 respectively; Fig. 3c,d). This difference could be accounted for by the six donors in the latter clinical group with a main neuropathological diagnosis of AD.

### Donors with LP show more widespread concomitant amyloid-β pathology

The level of AD-type pathology may be more closely related to the main neuropathological hallmarks than to the clinical phenotype. To examine this, donors were divided into four pathological diagnostic groups based on the presence of neuropathological hallmarks, i.e. LP, PSP-type tau pathology (PSP tau; including mixed PSP + LP), GCI pathology (including mixed GCI + LP) and other types of pathology. Compared to the LP group, disease onset was later in the PSP tau group (62 ± 11 vs. 66 ± 7 years, *p* = 0.006), age at death was younger in the GCI group (77 ± 8 vs. 66 ± 9 years, *p* < 0.001), and disease duration was significantly shorter in all three non-LP groups (15 vs. 8, 7 and 11 years; *p* < 0.001, *p* < 0.001 and *p* = 0.005 respectively). Dementia was more often present in the LP group than in the PSP tau and GCI group (55% vs. 27 and 7% respectively; *p* < 0.001 for both comparisons; Table 2).

**Table 2:** Demographics and clinical characteristics of donors with LP, PSP tau pathology, GCI pathology and other types of pathology

	LP	PSP tau pathology	GCI pathology	Other types of pathology
N	162	45	28	58
Sex *M*, n (%)	102 (63%)	28 (62%)	10 (36%)**	36 (62%)
Age at onset *y*, mean ± SD (range) < 50 / 50–75 / > 75 y, n (%)	62 ± 11 (32–88) 26/117/19 (16/72/12%)	66 ± 7 (51–84)** 0/40/5 (0/89/11%)	59 ± 9 (45–81) 3/23/2 (11/82/7%)	64 ± 12 (34–84) 7/43/8 (12/74/14%)
Age at death *y*, mean ± SD (range)	77 ± 8 (56–96)	74 ± 8 (57–90)	66 ± 9 (52–84)***	76 ± 11 (42–98)
Disease duration *y*, mean ± SD (range)	15 ± 7 (2–42)	8 ± 4 (3–23)***	7 ± 3 (3–16)***	11 ± 9 (0.5–41)**
Dementia, n (%)	89 (55%)	12 (27%)***	2 (7%)***	30 (52%)
Duration of dementia *y*, mean ± SD (range)	4 ± 3 (0–12)	2 ± 2 (0–6)**	1 ± 1 (0–1)	3 ± 2 (0–10)
Visual hallucinations, n/N (%)	93/122 (76%)	10/13 (77%)	5/7 (71%)	9/13 (69%)
Rest tremor, n/N (%)	112/134 (84%)	11/30 (37%)***	16/24 (67%)	29/37 (78%)
Clear response to dopaminergic therapy, n/N (%)	106/126 (84%)	6/26 (23%)***	11/24 (46%)***	10/26 (39%)***
Positive family history of parkinsonism, n/N (%)	31/80 (39%)	7/28 (25%)	3/17 (18%)	8/29 (28%)
APOE ε4 alleles, n/N (%)				
0	67/101 (66%)	23/39 (59%)	12/23 (52%)	33/43 (77%)
1	31/101 (31%)	15/39 (39%)	9/23 (39%)	9/43 (21%)
2	3/101 (3%)	1/39 (3%)	2/23 (9%)	1/43 (2%)

Groups are compared using analysis of covariance for continuous variables and chi-square tests for categorical variables. When significant, the LP group was compared to the other pathology groups using post-hoc tests for continuous variable and chi-square tests for categorical variables.* *p* < 0.05; ** *p* < 0.01; *** *p* < 0.001

Analysis of covariance including age at death was used to compare stages of AD-type pathology between groups (Fig. 3e-h). Thal amyloid-β phases were significantly different between donors with LP, PSP tau pathology, GCIs and other types of pathology, independent from age at death (F = 4.0, *p* = 0.009; Fig. 3f). Amyloid-β was more widespread in the LP group than in the GCI group (*p* = 0.013) and the group with other pathology (*p* = 0.015), but not the PSP tau group (*p* = 0.070). Braak NFT stage and CERAD neuritic plaque score were not higher in donors with LP compared to donors with other pathology, while controlling for age at death (F = 1.7, *p* = 0.16 and F = 1.7, *p* = 0.17 respectively; Fig. 3g and h).

### Amyloid-β pathology is related to dementia and neocortical LP

As dementia was more frequent in the LP group, we tested the role of the presence of dementia in the association between LP and Thal amyloid-β phases in a second analysis. Presence of dementia was associated with Thal amyloid-β phases in all donors (Spearman’s rho = 0.35, *p* = 0.002). Hierarchical clustering of all donors based on clinical features and pathological stages revealed that dementia was most closely related to stages of AD-type pathology, followed by age at death and age at onset (Supplementary Fig. 1). When including both age at death and presence of dementia in the model, the effect of type of pathology on Thal amyloid-β phases became smaller (F = 2.6, *p* = 0.057), while age at death and presence of dementia both contributed significantly to the model (F = 4.1, *p* = 0.045 and F = 14.1, *p* < 0.001 respectively).

Within the LP group, Thal amyloid-β phases were related to presence of dementia (Spearman’s rho = 0.22, *p* = 0.007), CERAD neuritic plaque score (rho = 0.71, *p* < 0.001), Braak NFT stage (rho = 0.29, *p* < 0.001), Braak α-synuclein stage (rho = 0.18, *p* = 0.037), and presence of neocortical LP (rho = 0.33, *p* < 0.001). Presence of dementia was significantly positively correlated to all α-synuclein and AD-type pathology stages (Braak α-synuclein stage Spearman’s rho = 0.26, presence of neocortical LP rho = 0.29, Thal amyloid-β phase rho = 0.22, Braak NFT stage rho = 0.29, CERAD neuritic plaque score rho = 0.29). Hierarchical clustering of 98 donors with LP with a complete clinicopathological dataset showed a close relation between dementia, Braak NFT stage and α-synuclein stages (Supplementary Fig. 2).

### MDS-PD criteria predict the presence of LP with high accuracy

**Fig. 4:**
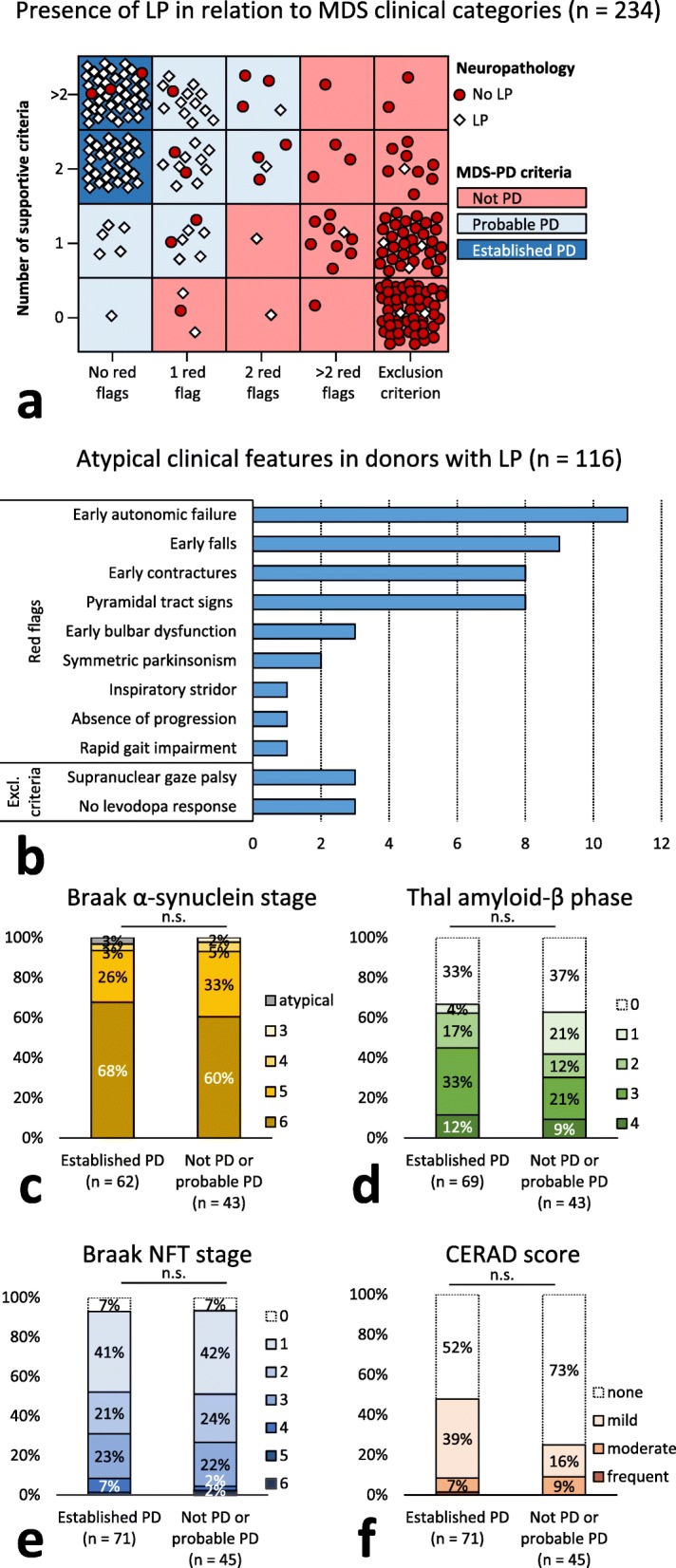
**a.** In 234 donors categorized according to the MDS-PD criteria [19], the number of supporting and atypical clinical features, i.e. red flags and exclusion criteria, correctly predicted the presence of LP in 89.3% of donors at a certainty level of ‘probable PD’, and in 79.5% of donors at a certainty level of ‘established PD’. **b**. The most common atypical clinical features in the donors with LP in our series (*n* = 116) were early autonomic failure and early falls. **c-f.** Among donors with LP, Braak α-synuclein stages (**c**), Thal amyloid-β phases (**d**), Braak NFT stages (**e**) and CERAD neuritic plaques scores (**f**) were not different in ‘established PD’ donors compared to ‘not PD’ and ‘probable PD’ donors

We determined the accuracy of the clinical diagnosis of PD to predict the presence of LP in our autopsy series by retrospectively applying the standardized MDS-PD criteria [19]. These criteria categorize patients as ‘not PD’, ‘probable PD’ or ‘established PD’ based on presence of supportive criteria, red flags and exclusion criteria. In the 234 donors with sufficient clinical information on these criteria, the certainty level of ‘probable PD’ predicted the presence of LP as the main neuropathological hallmark with a sensitivity of 90.5%, a specificity of 88.1%, and an overall accuracy of 89.3%. The certainty level of ‘established PD’ increased the specificity of the diagnosis of PD to 97.5%, at the cost of a decreased sensitivity (61.2%) and overall accuracy (79.5%; Fig. 4a).

### Pathological stages are similar between donors with LP in different MDS-PD clinical categories

Among 116 donors with LP, 11 donors (10%) were categorized as ‘not PD’, 34 donors (29%) as ‘probable PD’ and 71 donors (61%) as ‘established PD’ according to MDS-PD criteria [19]. Six donors with LP fulfilled exclusion criteria as defined by the MDS-PD criteria, including no response to adequate levodopa treatment (*n* = 3) and downward vertical supranuclear gaze palsy (*n* = 3). Atypical symptoms defined as ‘red flags’ according to the MDS-PD criteria were present in 37 out of 116 donors with LP (32%). The most common red flags in donors with LP included early severe autonomic failure (*n* = 11), early recurrent falls (*n* = 9), disproportionate anterocollis or limb contractures within first 10 years of disease (*n* = 8) and unexplained pyramidal tract signs (*n* = 8; Fig. 4b).

Donors categorized as ‘not PD’ or ‘probable PD’ were more often of the male sex (73% vs. 54%, *p* = 0.033), and had a significantly shorter disease duration than ‘established PD’ donors (13 ± 6 years vs. 17 ± 8 years; *p* = 0.003), but a similar age at death (76 ± 9 vs. 77 ± 8 years; *p* = 0.66). Presence of dementia at time of death was similar between groups (53% vs. 52%; *p* = 0.90; Table 3).

**Table 3:** Demographics and clinical characteristics in 116 donors with LP categorized according to MDS-PD criteria ^19^

	Established PD donors with LP	Not PD or probable PD donors with LP
N	71	45
Sex *M,* n (%)	38 (54%)	33 (73%)*
Age at onset *y,* mean ± SD (range)	60 ± 11 (32–78)	63 ± 12 (40–84)
Age at death *y,* mean ± SD (range)	77 ± 8 (56–90)	76 ± 9 (56–90)
Disease duration *y*, mean ± SD (range)	17 ± 8 (4–42)	13 ± 6 (3–31)**
Dementia, n (%)	37 (52%)	24 (53%)
Duration of dementia *y*, mean ± SD (range)	4 ± 3 (0–12)	4 ± 2 (0–7)
Visual hallucinations, n/N (%)	48/61 (79%)	25/36 (69%)
Rest tremor, n/N (%)	62/70 (89%)	31/42 (74%)*
Clear response to dopaminergic therapy, n/N (%)	68/71 (96%)	26/41 (63%)***
Positive family history of parkinsonism, n/N (%)	13/34 (38%)	14/26 (54%)
APOE ε4 alleles, n/N (%)		
0	28/45 (62%)	16/24 (67%)
1	15/45 (33%)	8/24 (33%)
2	2/45 (4%)	0/24 (0%)

P-values are shown for comparisons between ‘established PD’ and ‘not PD’ combined with ‘probable PD’ donors with LP using t-tests for continuous variables and chi-square tests for categorical variables. * *p* < 0.05; ** *p* < 0.01; *** *p* < 0.001

We tested whether different stages of α-synuclein and AD-type pathology were related to the clinical diagnosis of PD as defined by the MDS-PD criteria in donors with LP, using analysis of covariance with age at death as covariate. The Braak α-synuclein stage, Thal amyloid-β phase, Braak NFT stage and CERAD neuritic plaque score in donors with LP categorized as ‘not PD’ or ‘probable PD’ were not significantly different from the ‘established PD’ donors with LP (*p* = 0.23, *p* = 0.16, *p* = 0.91 and *p* = 0.23 respectively; Fig. 4c-f).

## Discussion

In brain donors that came to autopsy within the framework of the NBB, we assessed the neuropathological correlates of parkinsonian disorders. In summary, 85% of donors with a clinical diagnosis of PD showed LP, compared to 8% of donors with other clinical diagnoses. Furthermore, a wide variety of other types of pathology, such as GCIs, PSP tau pathology, vascular lesions and a rare tauopathy, were present in clinically-defined PD. Clinically-defined PD showed similar stages of AD-type pathology as clinically-defined MSA and PSP donors. However, donors with LP, i.e. pathologically-defined PD, showed more widespread amyloid-β pathology than donors with GCI pathology, i.e. pathologically-defined MSA, and more than donors with other types of pathology except for the pathologically-defined PSP group. The presence of LP was predicted with an accuracy of 89.3% by the MDS-PD criteria at a certainty level of ‘probable PD’. In donors with LP, Braak α-synuclein stages and stages of AD-type pathology were not different in donors categorized as ‘not PD’ or ‘probable PD’ compared to ‘established PD’ according to MDS-PD criteria.

The neuropathological correlates of parkinsonism have previously been evaluated in a number of autopsy series [2, 3, 5–7, 9, 10, 27]. These studies included donors with a final clinical diagnosis of PD [5, 27] or parkinsonian disorders [2, 3, 6, 7, 9, 10], and identified LP in 71–90% of clinical PD donors. MSA, PSP, vascular and AD pathology were other types of pathology that were most often found in clinical PD donors [2, 3, 6, 7, 9, 10, 27, 28]. In contrast to older studies [3, 5, 6, 27, 28], our series did not include any donors with post-encephalitic parkinsonism, reflecting the disappearance of this syndrome from clinical practice. In general, the pathological diagnoses in clinical PD donors in our autopsy series are in line with previous autopsy series from other settings and countries.

The common co-occurrence of LP in patients with PSP and MSA has been described before. Previous autopsy series in have identified LP in 31% (5/16) [29] and 22% (11/51) [15] of PSP donors. This is in line with the 18% mixed PSP + LP donors (8 out of all 45 pathologically-defined PSP donors) in our study. Neuronal α-synuclein pathology in MSA has been described before in detail by Cykowski et al. [30]. The authors found LB-like inclusions following the same stepwise anatomic progression as in Lewy body diseases in 9 out of 35 MSA donors (26%). Other autopsy series have reported LP in 0 out of 11 (0%) [31], 10 out of 94 (11%) [32] and 10 out of 44 (23%) [33] MSA cases. These varying percentages may arise from differences in case selection, regions used for examination, fixation and staining techniques, α-synuclein antibodies and varying definitions of LP or LB-like inclusions. Our study regarded all α-synuclein positive neuronal cytoplasmic inclusions following an anatomical distribution as in Lewy body diseases [26] as LP, which may explain the relatively high percentage of mixed MSA + LP donors (25% of all pathologically-defined MSA donors) in our autopsy series.

We hypothesized that concomitant AD-type pathology would be equally distributed among diagnostic groups. However, Thal amyloid-β phases were significantly higher in donors with LP than in donors with other pathological hallmarks, while controlling for the higher mean age at death of the donors with LP. This is in line with a recent autopsy series, where neocortical Lewy body disease was related to a 80% increase in amyloid-β pathology compared to a broad range of other neurodegenerative diseases [15]. Also, a positive correlation between insoluble α-synuclein levels and insoluble amyloid-β42 levels has been detected in PD brains [34]. It has been hypothesized that this may be caused by cross-seeding between α-synuclein and amyloid-β fibrils, for which there is accumulating evidence *in vitro *and *in vivo* [35, 36].

Furthermore, presence of dementia was significantly related to amyloid-β phases. The relation between amyloid-β and dementia in PD has been well-established in multiple autopsy series [16–18], as well as in an *in vivo *Pittsburgh compound B PET imaging study [37]. As donors with LP were more often diagnosed with dementia in our study, this largely explained the variance in amyloid-β phases between donors with LP and donors with other types of pathology. However, the high prevalence of dementia in the LP group compared to the other pathology groups likely reflects clinical differences between PD, PSP and MSA as reported by epidemiological studies [38, 39]. Therefore, correcting for the high proportion of demented donors in the LP group is likely not warranted.

Within the LP group, presence of dementia was more closely related to neurofibrillary tau pathology and neocortical LP than to amyloid-β pathology. Why both widespread amyloid-β pathology as well as dementia are more frequent in PD than in atypical parkinsonian disorders, in particular MSA, and whether this is the result of a direct interaction or synergistic effect of different pathologies in the PD brain, remains to be further studied.

A recent meta-analysis showed a pooled accuracy of 80.6% for the clinical diagnosis of PD in autopsy-based studies using pathological diagnosis as gold standard [8]. Therefore, the overall diagnostic accuracy of 89.3% for predicting presence of LP in the current study by the MDS-PD criteria can be regarded as relatively high. This may be due to the fact that this categorization was based on all clinical information during the entire course of disease. Over the course of the disease, the clinical phenotype may become more apparent, which is reflected by the longer disease duration in the ‘established PD’ donors with LP than the ‘not PD’ and ‘probably PD’ donors with LP. Therefore, on the one hand, our study may have overestimated the diagnostic accuracy in comparison to the accuracy that can be reached in clinical cohorts with a shorter follow-up period. However, in a clinical study for validation of the MDS-PD criteria, using expert diagnosis as gold standard, a diagnostic accuracy of 92.6% was found [40], suggesting that an equal high accuracy can be reached in clinical cohorts. On the other hand, PD patients with a more rapid progressive disease course may show more atypical clinical features than PD patients with a slower disease progression. Many ‘red flags’ from the MDS-PD criteria that were most common among donors with LP, are indeed related to a rapid disease progression, such as early severe autonomic failure, early recurrent falls and early contractures.

Interestingly, the specificity of the MDS-PD criteria for ‘established PD’ was 97.5% in our series. This observation indicates that the ‘established PD’ criteria identify patients with LP with a high level of certainty, at the cost of a lower sensitivity. These results indicate that the MDS-PD criteria for ‘established PD’ may aid in stratification of PD patients for clinical trials targeting α-synuclein or related proteins.

Among donors with LP, donors categorized as ‘not PD’ and ‘probable PD’ did not show a higher load of concomitant AD-type pathology or more widespread LP than ‘established PD’ donors. Therefore, we did not identify pathological correlates of the presence of atypical clinical features in the ‘not PD’ or ‘probable PD’ donors with LP. However, it should be noted that presence of atypical clinical features may be related to other types of concomitant pathology that were not assessed in this study, such as vascular lesions or TDP43 aggregates. Our results indicate that additional biomarkers are needed for characterizing underlying neuropathology in PD patients.

The strength of the current study is the combination of detailed clinical as well as neuropathological information in a relatively large autopsy series of donors with a broad range of parkinsonian disorders, which allows for a rigorous study of clinicopathological correlations in these disorders. Our study highlights the importance of brain banking to study discrepancies between clinical and neuropathological diagnosis, and to identify cases with rare causes of parkinsonian syndromes. Limitations of the current study include the retrospective nature of the clinical information, the fact that clinical diagnoses during life were made in different settings and partly by non-specialists, and a possible biased selection of patients that consent for brain donation to our brain bank [41].

## Conclusions

In conclusion, both the clinical diagnosis of PD and MDS-PD criteria accurately predicted the presence of LP in the NBB donors. Especially the MDS-PD criteria for ‘established PD’ may aid in identifying a group of PD patients with LP with a high level of certainty for inclusion in clinical trials. Importantly, classification of donors with LP based on the MDS-PD criteria only did not reflect differences in underlying neuropathology. Furthermore, amyloid-β was found to be associated with LP, suggesting a link between amyloid-β accumulation and LP formation.

## Supplementary information

**Additional file 1 Supplementary Figure 1.** When clustering all donors with a complete dataset (*n* = 164) based on clinical features and pathological stages, four clusters of donors can be distinguished, namely 1) a small cluster with high AD-type pathology, 2) a cluster with LP donors (except for 2 non-LP donors) with an early disease onset, a young age at death and relatively little AD-type pathology, 3) a cluster with almost all non-LP donors, and 4) a cluster with LP donors (except for 1 non-LP donor) with a late age at onset and a late age at death. **Supplementary Figure 2.** When clustering only the LP donors with a complete dataset (*n* = 98), Braak α-synuclein stages and neocortical LP are most closely related to Braak NFT stage and dementia.

## Data Availability

The datasets generated during and/or analysed during the current study are available from the corresponding author upon reasonable request and with permission of the Netherlands Brain Bank.

## Abbreviations

AD: Alzheimer’s disease
CBD: Corticobasal degeneration
CBS: Corticobasal syndrome
CERAD: Consortium to Establish a Registry for Alzheimer’s Disease
DLB: Dementia with Lewy bodies
FTD: Frontotemporal dementia
GCI: Glial cytoplasmic inclusion
LP: Lewy pathology
MDS: International Parkinson and Movement Disorder Society
MSA: Multiple system atrophy
NBB: Netherlands Brain Bank
NFT: Neurofibrillary tangles
PD: Parkinson’s disease
PSP: Progressive supranuclear palsy
PSP tau: Tau pathology typical for progressive supranuclear palsy
