# Supplementary material for: Neuropathological correlates of parkinsonian disorders in a large Dutch autopsy series
Source: Acta Neuropathol Commun. 2020 Mar 26;8:39. doi: 10.1186/s40478-020-00914-9 (PMC7098103; doi:10.1186/s40478-020-00914-9)
Supplement: Supplementary file 1 — Additional file 1 Supplementary Figure 1. When clustering all donors with a complete dataset (n = 164) based on clinical features and pathological stages, four clusters of donors can be distinguished, namely 1) a small cluster with high AD-type pathology, 2) a cluster with LP donors (except for 2 non-LP donors) with an early disease onset, a young age at death and relatively little AD-type pathology, 3) a cluster with almost all non-LP donors, and 4) a cluster with LP donors (except for 1 non-LP donor) with a late age at onset and a late age at death. Supplementary Figure 2. When clustering only the LP donors with a complete dataset (n = 98), Braak α-synuclein stages and neocortical LP are most closely related to Braak NFT stage and dementia. [file 40478_2020_914_MOESM1_ESM.docx]

**Supplementary Methods**

Cluster analysis was done on a dataset including all 164 donors with a complete set of the following data: sex, age at death, age at onset, disease duration, presence of dementia, MDS-PD diagnosis [3], neuropathological diagnosis, Thal amyloid-β phase [2], Braak NFT stage [2], CERAD neuritic plaque score [2] and Braak α-synuclein stage [1]. Hierarchical k-means clustering was done based on sex, age at death, age at onset, disease duration, presence of dementia and neuropathological stages.

In a second analysis, hierarchical clustering was done for all 98 LP donors with a complete dataset in a similar manner.


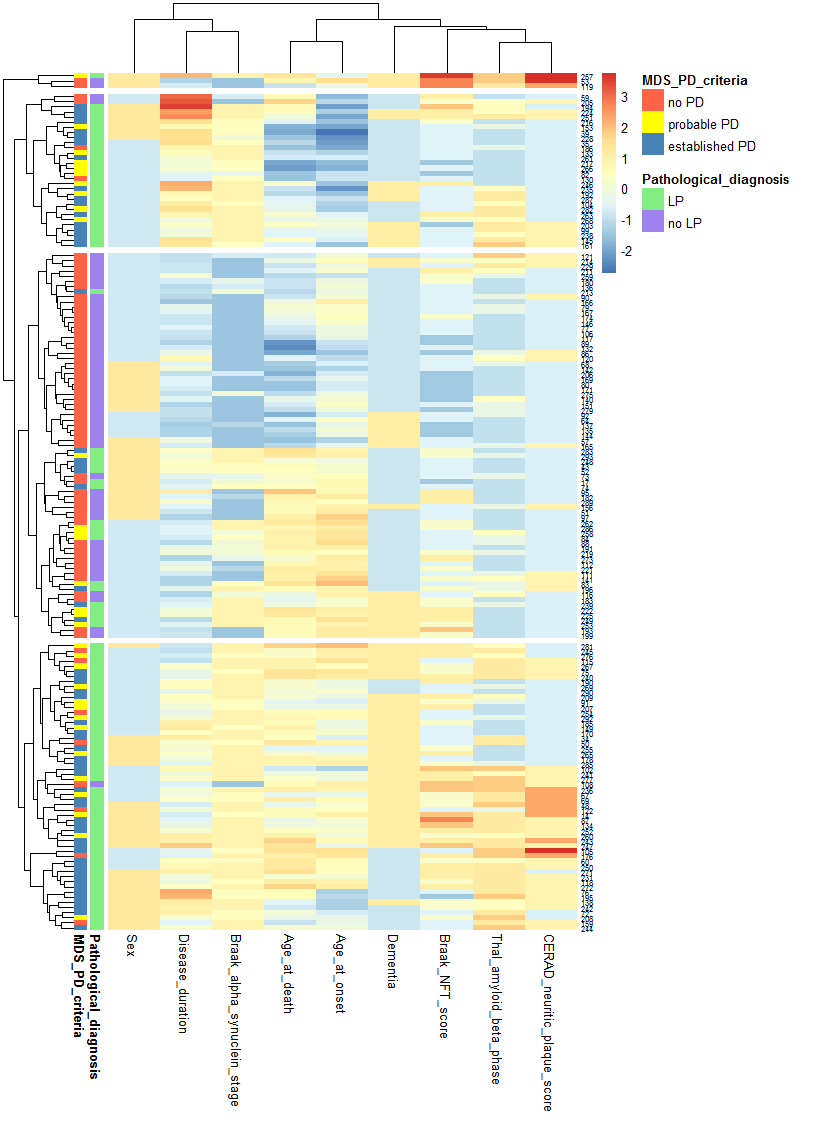


**Supplementary Figure 1.** When clustering all donors with a complete dataset (n = 164) based on clinical features and pathological stages, four clusters of donors can be distinguished, namely 1) a small cluster with high AD-type pathology, 2) a cluster with LP donors (except for 2 non-LP donors) with an early disease onset, a young age at death and relatively little AD-type pathology, 3) a cluster with almost all non-LP donors, and 4) a cluster with LP donors (except for 1 non-LP donor) with a late age at onset and a late age at death. The latter cluster can be split into a sub-cluster of mostly non-demented donors, and a sub-cluster of mostly demented donors. Dementia is most closely related to stages of AD-type pathology, followed by age at death and age at onset.

Sex: blue = male, orange = female; Dementia: blue = non-demented, orange = demented.


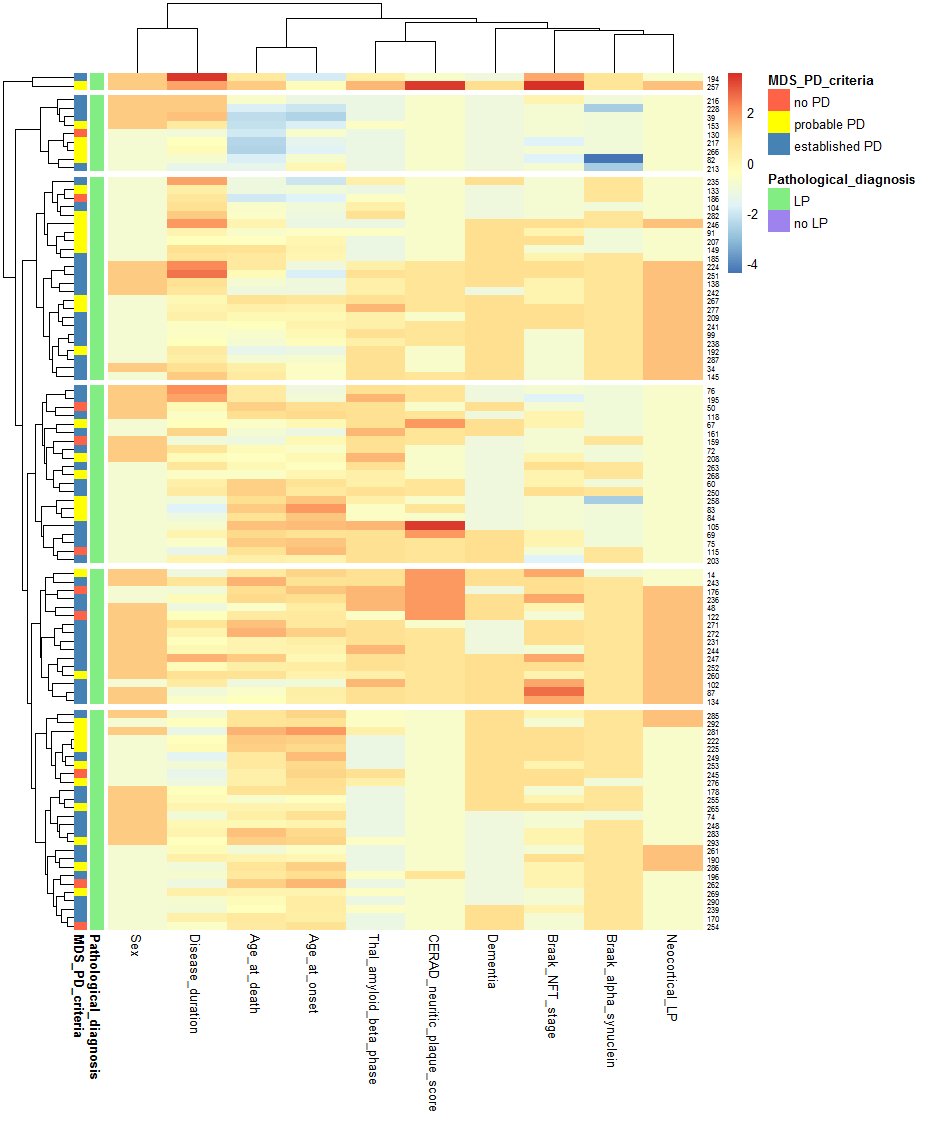


**Supplementary Figure 2.** When clustering only the LP donors with a complete dataset (n = 98), Braak α-synuclein stages and neocortical LP are most closely related to Braak NFT stage and dementia. Thal amyloid-β phase is most strongly related to CERAD neuritic plaque score.

Sex: yellow = male, orange = female; Dementia: blue = non-demented, orange = demented; Neocortical LP: yellow = no neocortical LP, orange = neocortical LP.

**References**

1 Alafuzoff I, Ince PG, Arzberger T, Al-Sarraj S, Bell J, Bodi I, Bogdanovic N, Bugiani O, Ferrer I, Gelpi Eet al (2009) Staging/typing of Lewy body related alpha-synuclein pathology: a study of the BrainNet Europe Consortium. Acta Neuropathol 117: 635-652 Doi 10.1007/s00401-009-0523-2

2 Hyman BT, Phelps CH, Beach TG, Bigio EH, Cairns NJ, Carrillo MC, Dickson DW, Duyckaerts C, Frosch MP, Masliah Eet al (2012) National Institute on Aging-Alzheimer's Association guidelines for the neuropathologic assessment of Alzheimer's disease. Alzheimer's & dementia : the journal of the Alzheimer's Association 8: 1-13 Doi 10.1016/j.jalz.2011.10.007

3 Postuma RB, Berg D, Stern M, Poewe W, Olanow CW, Oertel W, Obeso J, Marek K, Litvan I, Lang AEet al (2015) MDS clinical diagnostic criteria for Parkinson's disease. Mov Disord 30: 1591-1601 Doi 10.1002/mds.26424
